# Oil of Birch and Oil of Wintergreen

**Published:** 1890-08

**Authors:** 


### Oil of Birch and Oil of Wintergreen.

The *American Druggist*, March, 1890, says that oil of birch and oil of wintergreen were reported in August, 1889, by Messrs. Trimble and Schroeter to be physically and chemically identical, both consisting mainly of methyl salicylate with the addition of a hydrocarbon having a formula $C_{15}H_{24}$, together with smal quantities of benzoic acid and ethyl alcohol. They also stated that a sample of artificial wintergreen oil examined by them contained the properties of neither the natural oil nor of methyl salicylate. In reply to this Dr. Power reports the examination of a number of samples showing that natural wintergreen oil consists of methyl salicylate and 0.3 per cent. or less of lævogyrate terpene; that oil of birch, when pure, consisted simply of methyl salicylate, and is inactive towards polarized light; and that neither contains benzoic acid nor, so far as he has been able to satisfy himself, any ethylic alcohol. In the January number of the *Amer. Jour. of Pharm.*, Messrs. Trimble and Schroeter defend their position and combat some of these statements.

Willing to Trust Posterity.—A surgeon was called to see a rich but miserly patient, whom he found suffering from advanced cancer of the stomach. "And what are you going to charge me for treatment, doctor?" "Nothing; not a cent," was the reply. "Oh, how generous! How can I thank you?" said the patient. "No necessity for thanks, my friend, *your heirs* will see that I am paid."

